# Stretchable and Flexible Painted Thermoelectric Generators on Japanese Paper Using Inks Dispersed with P- and N-Type Single-Walled Carbon Nanotubes

**DOI:** 10.3390/s24092946

**Published:** 2024-05-06

**Authors:** Takumi Nakajima, Koki Hoshino, Hisatoshi Yamamoto, Keisuke Kaneko, Yutaro Okano, Masayuki Takashiri

**Affiliations:** Department of Materials Science, Tokai University, 4-1-1 Kitakaname, Hiratsuka 259-1292, Kanagawa, Japan

**Keywords:** single-walled carbon nanotubes, Japanese paper, painted thermoelectric generators, ink

## Abstract

As power sources for Internet-of-Things sensors, thermoelectric generators must exhibit compactness, flexibility, and low manufacturing costs. Stretchable and flexible painted thermoelectric generators were fabricated on Japanese paper using inks with dispersed p- and n-type single-walled carbon nanotubes (SWCNTs). The p- and n-type SWCNT inks were dispersed using the anionic surfactant of sodium dodecylbenzene sulfonate and the cationic surfactant of dimethyldioctadecylammonium chloride, respectively. The bundle diameters of the p- and n-type SWCNT layers painted on Japanese paper differed significantly; however, the crystallinities of both types of layers were almost the same. The thermoelectric properties of both types of layers exhibited mostly the same values at 30 °C; however, the properties, particularly the electrical conductivity, of the n-type layer increased linearly, and of the p-type layer decreased as the temperature increased. The p- and n-type SWCNT inks were used to paint striped patterns on Japanese paper. By folding at the boundaries of the patterns, painted generators can shrink and expand, even on curved surfaces. The painted generator (length: 145 mm, height: 13 mm) exhibited an output voltage of 10.4 mV and a maximum power of 0.21 μW with a temperature difference of 64 K at 120 °C on the hot side.

## 1. Introduction

Solution-based manufacturing processes, including inkjet printing, screen printing, vacuum filtering, and drop casting, are essential for many types of functional thin-film devices because they enable the development of low-cost, scalable, and vacuum-free manufacturing processes [1,2,3,4,5,6,7,8,9,10]. Major functional thin-film devices that use solution-based manufacturing processes include photovoltaic cells, sensors, transistors, and thermoelectric devices. In particular, perovskite photovoltaic cells fabricated via several solution-based manufacturing processes have been the most successful examples in recent years [11,12,13,14,15]. The emergence of perovskite photovoltaic cells has caused significant changes in the solar-cell industry, which was previously dominated by silicon.

Other functional thin-film devices can potentially make significant advances in society. One such device is a thermoelectric generator, which converts thermal energy into electricity [16,17,18,19,20]. Conventional thin-film thermoelectric generators are typically fabricated using inorganic materials such as bismuth telluride-based alloys through dry processes such as sputtering, vacuum evaporation, and chemical vapor deposition [21,22,23,24,25,26]. In recent years, the development of lightweight and flexible thermoelectric generators as independent power sources for Internet-of-Things (IoT) sensors, such as wearable devices and wireless sensor nodes, has advanced rapidly [27,28,29,30,31,32,33,34]. The use of thermoelectric generators as a power source for IoT sensors obviates the necessity to replace sensor batteries and wiring, thereby greatly expanding the places where IoT sensors can be used. Therefore, thermoelectric materials and manufacturing processes that can replace traditional thin-film thermoelectric generators must be developed.

Promising thin-film thermoelectric generators have been fabricated from organic materials or single-walled carbon nanotubes (SWCNTs) via solution-based manufacturing processes [35,36,37,38,39]. In particular, SWCNTs are known for their excellent flexibility, heat resistance, and simplicity of thin-film fabrication despite their inferior thermoelectric performance compared to inorganic thermoelectric materials [40,41,42]. Recent studies have shown that stable n-type SWCNT films can be obtained using several types of doping materials [43,44,45]. Based on these studies, it is now possible to obtain both p- and n-type SWCNT films and fabricate p–n junction SWCNT thermoelectric generators [46,47,48]. In our previous studies, n-type SWCNT films with ultra-long stability for over two years were developed using a cationic surfactant as a dopant [49], and several p–n junction SWCNT thermoelectric generator structures were developed using vacuum filtration [50]. During manufacturing, the completed SWCNT films were cut to the desired size and attached to the substrate using double-sided tape. To reduce manufacturing costs and time, as well as increase the convenience of using p–n junction SWCNT thermoelectric generators, innovative manufacturing processes and generator structures are required.

This study presents thin-film thermoelectric generators on paper painted with inks dispersed in p- and n-type SWCNTs. The painting method using the inks could be expanded into a large-scale printing technology method, thereby enabling mass production of thin-film thermoelectric generators, which could significantly reduce production costs and improve cost-effectiveness. Paper was used as a substrate for the generators for three reasons. First, the inks easily soak into the paper, and p–n junction SWCNT thermoelectric generators can be fabricated by painting the inks into areas with p-type and n-type properties [51,52]. Second, compared to the thermal conductivity of SWCNT films, this study employs a low thermal conductivity, which can cause temperature differences in the generators [53,54]. Third, thermoelectric generators that are flexible, inexpensive, and recyclable have been realized on paper [55,56,57,58,59]. However, paper is unsuitable for use in high-temperature environments, as it begins to darken when exposed to temperatures above 150 °C. Therefore, thin-film thermoelectric generators painted on paper using inks are useful for low- to mid-temperature applications, where most IoT sensors work [60]. The fabricated thermoelectric generators are stretchable and flexible, similar to accordions, making them suitable for use in spaces of varying sizes and curvatures. We evaluated the structural and thermoelectric properties of painted p- and n-type SWCNT layers and measured the performance of the painted generator by creating a temperature difference.

## 2. Materials and Methods

**Figure 1:**
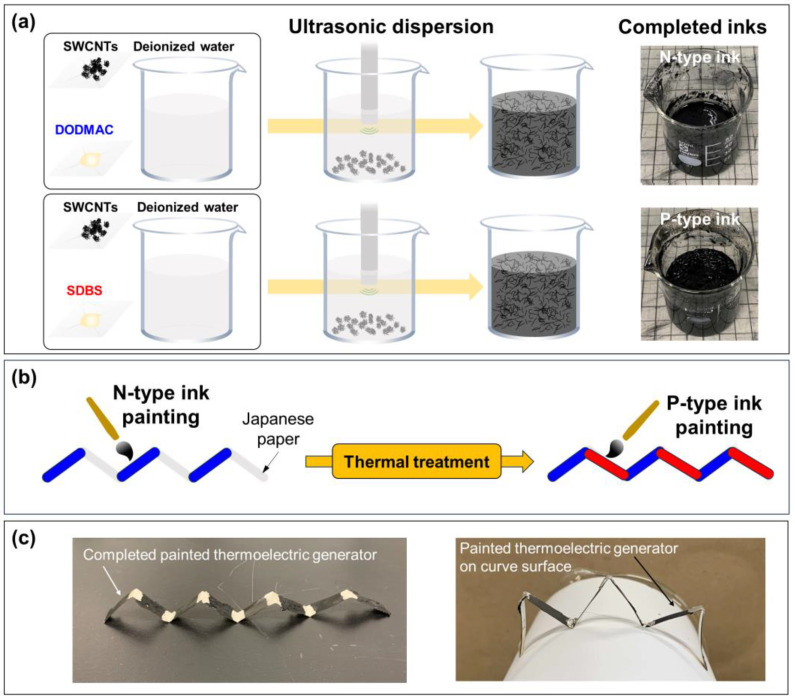
Fabrication process of painted thermoelectric generators on Japanese paper. (**a**) Ultrasonic dispersion of n- and p-type SWCNT inks, (**b**) painting n- and p-type SWCNTs on Japanese paper, and (**c**) completed painted generator on a curved surface with various shrink and expand conditions.

Figure 1 shows the fabrication of the p–n junction SWCNT thermoelectric generators. In Figure 1a, the starting materials used in this study were SWCNTs synthesized using the super-growth method (SG-CNTs) (ZEONANO SG101, ZEON Co., Tokyo, Japan) [61]. An n-type ink was prepared by adding 80 mg of SG-CNT powders and 400 mg of dimethyldioctadecylammonium chloride (DODMAC) (Fujifilm Wako Pure Chemical, Osaka, Japan) as a cationic surfactant in 40 mL of deionized water, resulting in a concentration of 0.2 wt% of SWCNT and 1.0 wt% of DODMAC. The ink was completely dispersed using an ultrasonic homogenizer (Branson Sonifier SFX 250, Emerson, St. Louis, MO, USA) at 70% output power (nominal 200 W) for 60 min in an ice bath. The dispersion condition, particularly the dispersion output power, was determined through our preliminary experiments. In these experiments, when the dispersion output power was low, the dispersibility of the SWCNTs was low, resulting in thick SWCNT bundles. Conversely, when the dispersion output power was high, the dispersibility of the SWCNTs increased, but the SWCNTs were damaged. Therefore, in this study, the dispersion output power was set to a medium level (70%) to achieve an optimal balance between the two aforementioned conditions. Consequently, the n-type ink with a density of 0.83 g/mL and a pH of 4.0 was prepared. Conversely, a p-type ink was prepared by adding 80 mg of SG-CNT powders and 200 mg of sodium dodecylbenzene sulfonate (SDBS) (Tokyo Chemical Industry, Tokyo, Japan) as an anionic surfactant in 40 mL of deionized water, resulting in a concentration of 0.2 wt% of SWCNT and 0.5 wt% of SDBS. The dispersion condition of the p-type ink, prepared using an ultrasonic homogenizer, was identical to that of the n-type ink. Consequently, the p-type ink with a density of 0.77 g/mL and a pH of 4.1 was prepared.

In Figure 1b, Japanese paper (Kami-4AW, Maruai, Kyoto, Japan) was used as the substrate because it is very strong and has a relatively long life compared to conventional paper. Additionally, Japanese paper is less expensive than polymer films and does not emit harmful gases when burned. The paper for the thermoelectric generator was approximately 100 μm thick, 10 mm wide, and 240 mm long. No additional treatment was performed on the Japanese paper used in this study to enhance the ink’s stability. Assuming that the generators would be placed on the curved surface of tubes with typical diameters ranging from 50 mm to 200 mm, the Japanese paper was folded in a mountain-valley pattern at 30 mm intervals. Using tweezers and brushes, n-type ink was painted on the Japanese paper at specific regions: 0–30 mm, 60–90 mm, 120–150 mm, and 180–210 mm from the left edge. To maintain a consistent application of SWCNT ink in each region, approximately 0.6 g of SWCNT dispersion was absorbed into the brush and applied uniformly to the Japanese paper until the dispersion in the brush was nearly depleted. This process was repeated three times for each area. The thickness of the n-type region, including the paper thickness, ranged from 150 μm to 240 μm. To develop n-type properties with ultra-long air stability, the thermoelectric generators were subjected to heat treatment in an electric furnace with a mixture of argon (95%) and hydrogen (5%) gases at atmospheric pressure [49]. The heat treatment temperature was set at 150 °C, and the treatment duration was 1 h. After heat treatment, p-type ink was painted on the Japanese paper in specific regions: 30–60 mm, 90–120 mm, 150–180 mm, and 210–240 mm from the left edge, using the same procedure and amount of SWCNT dispersion as that used for n-type ink painting. The thickness of the p-type region, including the paper thickness, ranged from 120 μm to 150 μm. The difference in film thickness between the n- and p-type SWCNT layers can be attributed to the differential absorption of the inks into the paper, despite the nearly equal amount of paint applied to both types of inks. The details are presented in the next section. We ensured that both types of SWCNT layers adhered well to the Japanese paper. To enhance the electrical conductivity of the generators, a silver paste was painted at the interfaces between the n- and p-type SWCNT layers. Subsequently, the copper electrodes were connected to both ends of the generator using silver paste. Photographs of the complete generator are shown in Figure 1c. The generator can be stretched or shrunk on a curved surface; the valleys of the generator are in contact with the curved surface.

To investigate the structural and thermoelectric properties of the p- and n-type SWCNT layers, each ink was painted on Japanese paper with a size of 40 mm^2^. The paper specifications, including the thickness and conditions of painting and heat treatment, were the same as those used for the p–n junction SWCNT thermoelectric generators. The microstructure and crystallinity of the p- and n-type SWCNT layers were analyzed using field-emission scanning electron microscopy (FE-SEM, Hitachi, S-4800, Victoria, BC, Canada) and Raman spectroscopy (HORIBA, XploRA, Kowloon, Hong Kong). The in-plane Seebeck coefficient *S* and electrical conductivity *σ* were measured using the ZEM-3 method (Advance Riko, ZEM-3, Kanagawa, Japan) in a helium atmosphere over a temperature range of 30 °C to 150 °C. The in-plane power factor *PF*, which is a crucial parameter for evaluating thermoelectric performance, was calculated using the equation *PF* = ${\sigma S}^{2}$.

## 3. Results and Discussion

### 3.1. Structural Properties of SWCNT Layers

**Figure 2:**
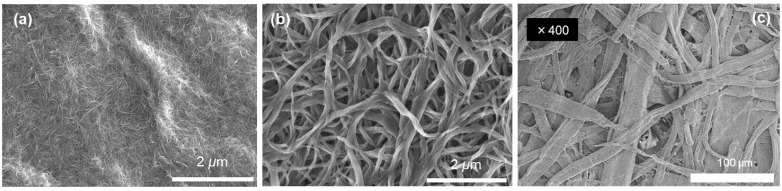
Surface morphology of (**a**) p-type and (**b**) n-type painted SWCNT layers on Japanese paper, and (**c**) Japanese paper alone (low magnification).

The microstructures of the p- and n-type SWCNT layers observed using FE-SEM are shown in Figure 2. The surface morphologies of the SWCNT layers differed significantly between the p- and n-types. As shown in Figure 2a, the p-type SWCNT layer consists of several entangled SWCNT bundles with very small diameters (tens of nanometers). As shown in Figure 2b, the n-type SWCNT layer is composed of entangled SWCNT bundles with relatively large diameters (tens of micrometers) compared to that of the n-type layer. The difference in bundle diameters between the two types of layers suggests that SDBS, rather than DODMAC, improved the dispersibility of the SWCNTs. Therefore, the difference in dispersibility is believed to affect the thickness of the layer. The relationship between the bundle diameter and dispersibility of SWCNTs has been reported for different dopants [62,63]. In the p-type SWCNT layer, the ink was easily absorbed by the Japanese paper because of its very small bundle diameter compared with the fabric diameter of the paper, as shown in Figure 2c. In contrast, in the n-type layer, the ink did not penetrate deeply into the Japanese paper because of its relatively large bundle diameter compared to the p-type SWCNT layer. Consequently, the p-type SWCNT layer is thinner than the n-type SWCNT layer.

**Figure 3:**
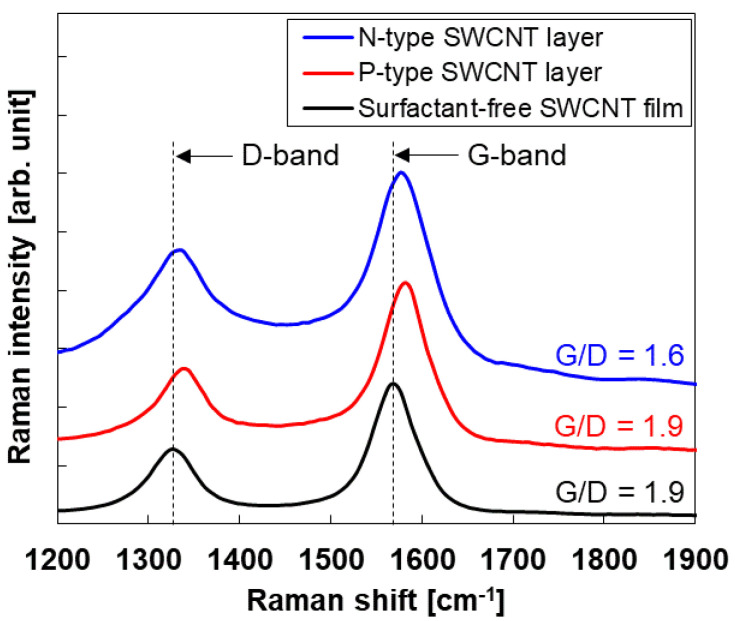
Raman spectra of p- and n-type painted SWCNT layers on Japanese paper and surfactant-free SWCNT film.

The Raman spectra of the p- and n-type SWCNT layers are shown in Figure 3. For comparison, we added the Raman spectrum of the surfactant-free SWCNT film prepared using the same starting material (SG-CNT powders) via vacuum filtration, based on our previous report [64]. Distinct Raman shifts associated with the SWCNTs are observed, including the G-band at ≈1590 cm^−1^ and the D-band at approximately 1340 cm^−1^. The degree of crystallinity of the SWCNTs was determined from the G/D ratio, which represents the intensity ratio of the G and D bands. The G/D ratios of the p- and n-type SWCNT layers and surfactant-free SWCNT film were 1.9, 1.6, and 1.9, respectively. These trends suggest that the crystallinity of the SWCNTs remains largely unaffected by the different doping conditions. However, downshifts of the G- and D-bands were observed in both the p- and n-type SWCNT layers with almost identical magnitudes. The downshift of the bands occurred because of the effective surfactant doping. Similar phenomena have been observed in other doped materials [65].

### 3.2. Thermoelectric Properties of SWCNT Layers

**Figure 4:**
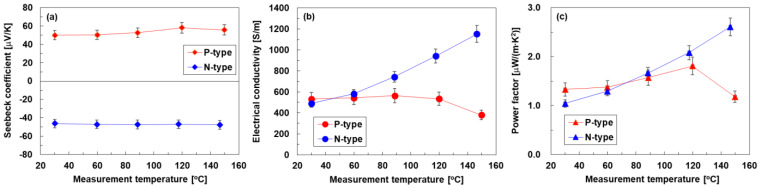
Temperature-dependent in-plane thermoelectric properties of p- and n-type painted SWCNT layers on Japanese paper: (**a**) Seebeck coefficient, (**b**) electrical conductivity, (**c**) power factor.

Figure 4 shows the in-plane thermoelectric properties of p- and n-type SWCNT layers measured at temperatures ranging from 30 °C to 150 °C. In Figure 4a, the Seebeck coefficients of p- and n-type SWCNT layers measured at 30 °C were 50 μV/K and −46 μV/K, respectively. Both layers of SWCNTs maintained mostly consistent values even as the temperature increased from 30 °C to 150 °C.

Figure 4b shows the electrical conductivities of the SWCNT layers at different temperatures. Notably, the electrical conductivity was calculated using the layer thickness, which does not account for the ink soaked in the Japanese paper. In p-type SWCNT layers, the electrical conductivity was maintained at a similar value of 550 S/m temperatures below 120 °C. When the temperature was increased to 150 °C, the electrical conductivity decreased to 380 S/m. In contrast, the electrical conductivity of the n-type SWCNT layers at 30 °C was comparable to that of the p-type SWCNT layers at the same temperature. However, the electrical conductivity of n-type SWCNT layers increased linearly as the temperature was raised from 30 °C to 150 °C. The electrical conductivity of the n-type SWCNT layer at 150 °C was 1153 S/m, which was 2.4 times higher than that at 30 °C.

To investigate the factors that cause differences in the temperature dependence of the electrical conductivity of the p- and n-type SWCNT layers, the thermoelectric properties of a surfactant-free SWCNT film were measured, which is the same sample used for Raman spectroscopy (Figure 3). The temperature dependence of the thermoelectric properties of the fabricated surfactant-free SWCNT films is provided in the Supplementary Information (Figure S1). The surfactant-free SWCNT film showed an increase in electrical conductivity and a decrease in the p-type Seebeck coefficient as the temperature was raised from 30 °C to 150 °C. These observations indicate that the surfactant-free SWCNT film exhibits semiconducting behavior; that is, the interband transitions of holes occur by absorbing thermal energy in the far-infrared region. This was consistent with a previous report on SWCNT films with similar G/D ratios [66]. In the n-type SWCNT layer, the electrical conductivity increased, whereas the Seebeck coefficient remained relatively stable as the measurement temperature increased. These phenomena occurred because the carrier concentration remained unchanged, whereas the mobility increased as the temperature increased. Therefore, cationic surfactant components are expected to adsorb tightly onto the SWCNT surface and enhance the electronic conduction of the entire SWCNT bundle without interband electron transitions. To further enhance the electrical conductivity of the n-type SWCNT layers, an effective approach is to use SWCNTs with a high G/D ratio. In contrast, in the p-type SWCNT layers, the temperature dependence of the electrical conductivity is affected by the change in the anionic surfactant component adsorbed on the SWCNT surface in the high-temperature region, which inhibits current flow and reduces hole mobility.

To confirm the change in the anionic surfactant on the SWCNT surface, we remeasured the thermoelectric properties of the p-type SWCNT layer in the same sample, as shown in the Supplementary Information (Figure S2). Even after re-measuring the thermoelectric properties of the p-type SWCNT layer at 30 °C, the values remained low. Thus, it can be inferred that the anionic surfactant component changes at temperatures exceeding 150 °C, which corresponds to our previous report [67]. Therefore, we are considering using anionic surfactants other than SDBS, such as sodium dodecyl sulfate (SDS) that can stabilize the p-type properties of the SWCNT layer from room temperature to high temperatures, specifically the nonburning temperature of paper. Currently, when evaluating the performance of paintable SWCNT thermoelectric generators, the upper-temperature limit is set at 120 °C.

In Figure 4c, the power factors of p- and n-type SWCNT layers measured at 30 °C were 1.3 μW/(m·K^2^) and 1.0 μW/(m·K^2^), respectively. The temperature-dependent power factor of both types of SWCNT layers was mainly reflected in the electrical conductivity, owing to the small variation in the Seebeck coefficient with temperature. The power factor of the n-type SWCNT layer at 150 °C was 2.6 μW/(m·K^2^), which was 2.2 times higher than that of the p-type SWCNT layer at the same temperature.

### 3.3. Performance of Thin-Film Thermoelectric Generators

**Figure 5:**
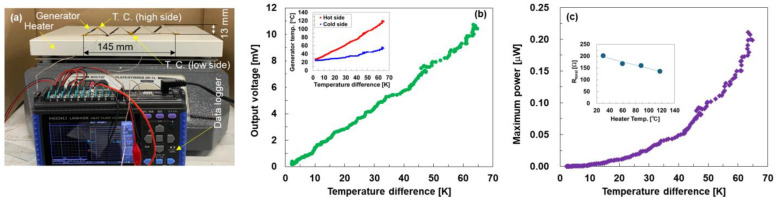
(**a**) Photograph of performance measurement of painted thermoelectric generator on Japanese paper. (**b**) Output voltage and (**c**) maximum power of the generator as a function of temperature difference. Insets show (**b**) relationship between generator temperatures and temperature difference and (**c**) relationship between heater temperature and total resistance of generator.

The system used to measure the performance of the painted thermoelectric generator is shown in Figure 5a. The generator was folded to a length of 145 mm and positioned with the valleys in contact with the heater. To take advantage of the flexibility of the generator, it was not fixed to the heat source using adhesives. The height was 13 mm owing to the folding condition. Two copper wires for voltage measurements and two thermocouples for temperature measurements at high and low temperatures were attached to the generator, and the opposite sides of the copper wires and thermocouples were connected to a data logger (GRAPHTEC, GL240).

Figure 5b shows the output voltage of the painted thermoelectric generator as a function of temperature difference. The inset in this figure illustrates the correlation between the temperature difference and the temperatures on the hot and cold sides of the generator. When the temperature of the generator’s hot side increased steadily from 25 °C to 120 °C at a rate of approximately 0.5 K per second, the temperature of the cold side also increased from 25 °C to 56 °C. Consequently, the temperature difference increased from 0 to 64 K. The output voltage of the generator increased linearly with the temperature difference. When the temperature difference reached 64 K while the hot side temperature was 120 °C, the output voltage was 10.4 mV. This value is insufficient for powering IoT sensors, which require a minimum output voltage of 20 mV [68]. When the Seebeck coefficients of the p- and n-type SWCNT layers were directly applied to the generator performance, as shown in Figure 4a, the output voltage was expected to be approximately 24 mV. Therefore, the actual generator performance was only approximately 50% of the expected value. This phenomenon is due to the fact that part of the generator valley was not in firm contact with the heater, and only a small temperature difference occurred inside the generator in this area. Therefore, the adhesion between the generator and heat source should be improved before being used in the power supplies of IoT sensors.

Figure 5c shows the maximum power of the painted thermoelectric generator as a function of temperature difference. The maximum power *P_max_* is expressed as *P_max_* = *V_oc_*^2^/4*R_total_*, where *V_oc_* and *R_total_* represent the output voltage and measured total resistance of the generator, respectively, and the total resistance is the sum of the SWCNT layers, electrodes, and contact resistances. The total resistance of the generator could not be determined by measuring the output voltage and temperature of our system. Therefore, the total resistance was measured individually by varying the temperature of the heater, as shown in the inset. The total resistance decreased from 200 to 135 Ω as the heater temperature increased from 30 °C to 120 °C, primarily due to the increase in the electrical conductivity of the n-type SWCNT layers. The maximum power of the generator increased quadratically when the temperature difference increased. The maximum power at a temperature difference of 64 K is 0.21 μW.

Table 1 compares the effectiveness of thermoelectric generators painted on Japanese paper with SWCNT ink to that of flexible thermoelectric generators that use similar SWCNT inks. The only differences between the two are the generator structure and the manufacturing process of the SWCNT films. The reference generator used SWCNT films prepared on polyimide sheets using the drop-cast method [49]. When the temperature differences in the generators were nearly equal, the output voltage and maximum power of the painted thermoelectric generator on Japanese paper were approximately half those of a drop-cast thermoelectric generator on a polyimide sheet. Furthermore, this work compares the maximum power of the generator to that of the flexible thermoelectric generator comprising inorganic materials of n-type Bi_2_Te_3_ and p-type Sb_2_Te_3_ thin films, which exhibit high thermoelectric properties near 300 K [22]. The generator comprising inorganic materials with a similar film length (*L* = 26 mm) exhibited a maximum power of 0.11 µW at a temperature of 40 K. This value is approximately two times larger than that of the thermoelectric generators in this study at the same temperature difference. Therefore, even though there is still room for improvement in the performance, such as the thermal durability of p-type SWCNT films and the adhesion condition of the heat source, we demonstrated that electricity can be generated in stretchable and flexible thermoelectric generators fabricated on Japanese paper painted with inks dispersed with p- and n-type SWCNTs. By improving the generator’s performance, it can be used as a power source for IoT sensors in narrow and curved locations such as automobiles and pipes for hot water and oils by using the advantages of their stretchable and flexible characteristics. Furthermore, the stability and reliability of the painted thermoelectric generator are currently being evaluated, as they are important factors for commercialization.

Finally, we discuss the environmental impact and sustainability aspects of using SWCNTs in the fabrication of thermoelectric generators. The manufacturing process in this study does not use equipment that requires high electric power, which reduces CO_2_ emissions. Regarding disposal, the device can be incinerated after use, and no toxic gases are generated. Furthermore, the waste can be recycled back into the original paper and SWCNTs, as follows. The generators are cut into small pieces, and water and chemicals are added to them. The pieces are then stirred to produce a dispersion of SWCNTs and paper fibers. By filtering them, the SWCNTs and paper fibers can be separated and reused.

## 4. Conclusions

Stretchable and flexible painted thermoelectric generators were prepared on Japanese paper using inks dispersed in p- and n-type SWCNTs. Japanese paper is suitable as a substrate for generators because it is strong and has a relatively long life compared to conventional paper. The p- and n-type SWCNT inks were dispersed with the anionic surfactant of SDBS and the cationic surfactant of DODMAC, respectively. The p- and n-type SWCNT inks were painted on the Japanese paper to prepare the p- and n-type SWCNT layers, respectively. The Seebeck coefficients for both p- and n-type SWCNT layers were almost constant for the entire temperature range (30 °C–150 °C), respectively. However, the electrical conductivity of the n-type SWCNT layer exhibited an increase with temperature, while that of the p-type SWCNT layer exhibited a constant value below 120 °C and a decrease at 150 °C. When the thermoelectric generators were prepared, the Japanese paper was folded into a mountain-valley structure, and p- and n-type inks were applied to it to form a striped pattern. Similar to accordions, the resulting generators can be placed in tight spaces by shrinking and in wide spaces by expanding, even though the surfaces are curved. To measure the performance of the generator, it was folded to a length of 145 mm and a height of 13 mm and placed on a heater. At a temperature difference of 64 K, the generator exhibited an output voltage of 10.4 mV and a maximum power of 0.21 μW. Therefore, we demonstrated the operation of stretchable and flexible thermoelectric generators using Japanese paper and SWCNT ink-painting processes.

## Figures and Tables

**Table 1:** Comparison of SWCNT thermoelectric generators (TEGs) painted on Japanese paper and drop-casted on polyimide sheet.

Structure of Generator	Painted TEG on Japanese Paper	Drop-Casted TEG on Polyimide Sheet
*V_oc_* [mV]	10.7	24
*P_max_* [μW]	0.21	0.4
Number of pair	4	4
SWCNT powder	SG-CNT	SG-CNT
Surfactant (P-type)	SDBS	No use
Surfactant (N-type)	DODMAC	DODMAC
Reference	This work	[49]

## Data Availability

Research data can be shared by M.T., if requested.

## Supplementary Materials

The following supporting information can be downloaded from https://www.mdpi.com/article/10.3390/s24092946/s1. Figure S1: Temperature dependence of the in-plane thermoelectric properties of surfactant-free SWCNT films. (a) Seebeck coefficient, (b) electrical conductivity, and (c) power factor; Figure S2: Temperature dependence of in-plane thermoelectric properties of p-type SWCNT layers (reproducibility measurement). (a) Seebeck coefficient, (b) electrical conductivity, and (c) power factor.
