# Supplementary material for: Stretchable and Flexible Painted Thermoelectric Generators on Japanese Paper Using Inks Dispersed with P- and N-Type Single-Walled Carbon Nanotubes
Source: Sensors (Basel). 2024 May 6;24(9):2946. doi: 10.3390/s24092946 (PMC11086293; doi:10.3390/s24092946)
Supplement: Supplementary file 1 [file sensors-24-02946-s001.zip › sensors-2958274-supplementary.pdf]

Stretchable and Flexible Painted Thermoelectric Generators on Japanese Paper Using Inks Dispersed with P- and N-Type Single-Walled Carbon Nanotubes

Takumi Nakajima, Koki Hoshino, Hisatoshi Yamamoto, Keisuke Kaneko, Yutaro Okano and Masayuki Takashiri *

Department of Materials Science, Tokai University, 4-1-1 Kitakaname, Hiratsuka, Kanagawa 259-1292, Japan
* Correspondence: takashiri@tokai.ac.jp

Supplementary Information

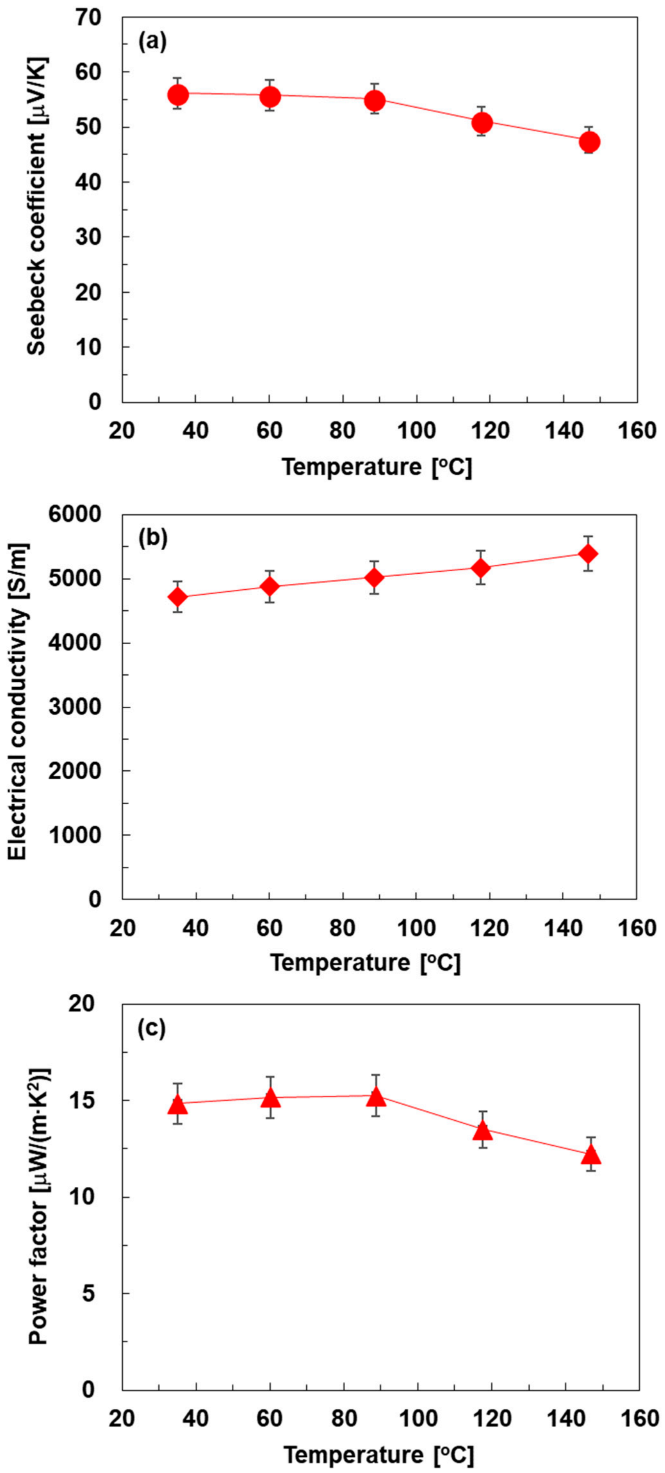

Figure S1: Temperature dependence of the in-plane thermoelectric properties of surfactant-free SWCNT films. (a) Seebeck coefficient, (b) electrical conductivity, and (c) power factor.

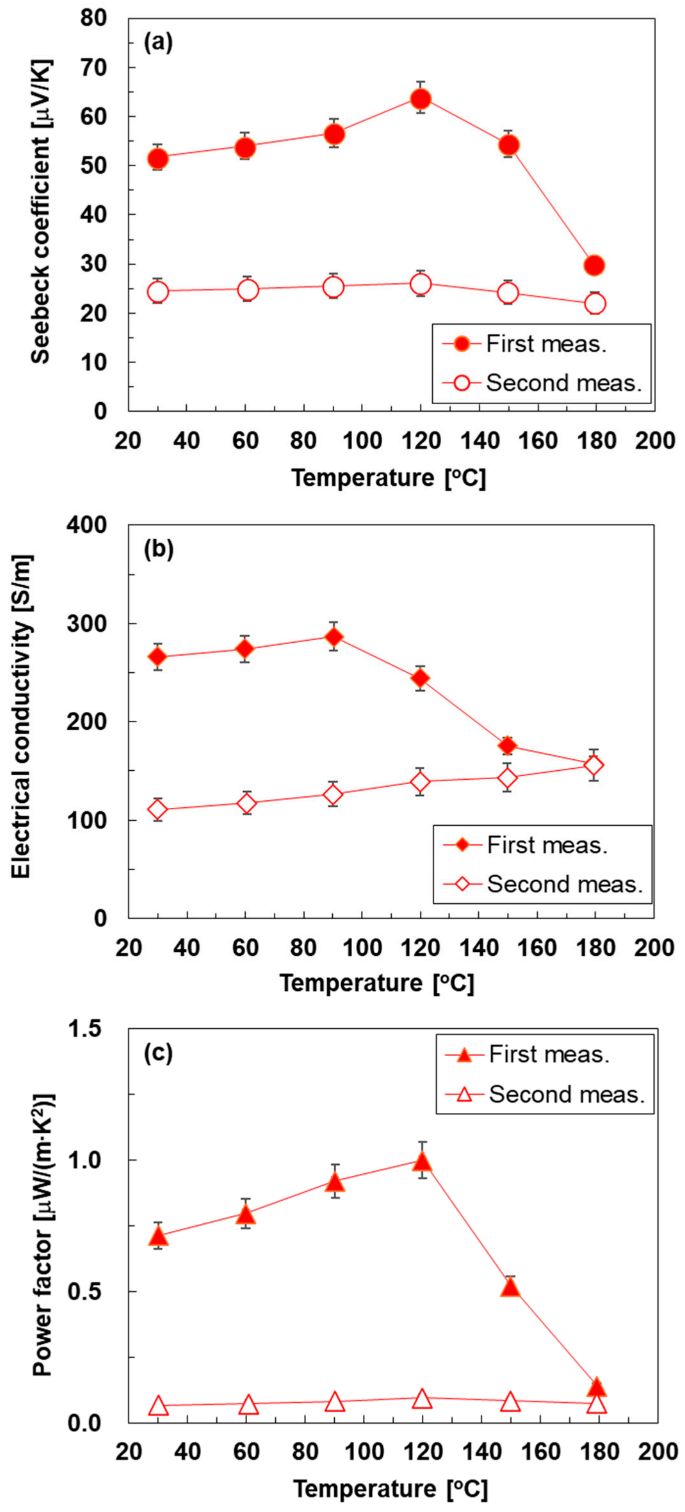

Figure S2: Temperature dependence of in-plane thermoelectric properties of p-type SWCNT layers (reproducibility measurement). (a) Seebeck coefficient, (b) electrical conductivity, and (c) power factor.
